# Identifying core habitats and corridors of a near threatened carnivore, striped hyaena (*Hyaena hyaena*) in southwestern Iran

**DOI:** 10.1038/s41598-022-07386-y

**Published:** 2022-03-02

**Authors:** Kamran Almasieh, Alireza Mohammadi, Rahim Alvandi

**Affiliations:** 1 Department of Nature Engineering, Agricultural Sciences and Natural Resources University of Khuzestan, Mollasani, Iran; 2 Department of Environmental Science and Engineering, Faculty of Natural Resources, University of Jiroft, Jiroft, Iran; 3 Khuzestan Provincial Office of the Department of Environment, Ahvaz, Iran

**Keywords:** Ecology, Zoology

## Abstract

Conservation of large carnivores requires preservation of extensive core habitats and linkages among them. The goal of this study was to identify core habitats and corridors by predicting habitat suitability (an ensemble approach), and calculating resistant kernel and factorial least-cost path modeling for a relatively unknown carnivore, the striped hyaena in Khuzestan area in southwestern Iran. We used the procedure of spatial randomization test to evaluate the coincidence of striped hyaena road crossing with the predicted corridors. The results revealed that elevation, distance to conservation areas, categorical climate and grasslands density were the most influential variables for predicting the occurrence of the striped hyaena in the study area. In the estimated dispersal distance of 70 km, four core habitats were identified. The largest core habitat was located in the northeast of the study area with the highest connectivity contribution. Only about 12% and 1.5% of core habitats and corridors were protected by conservation areas, respectively. Predicted corridors, crossed by roads represented a high risk for striped hyaenas. Adaptive management plan throughout the landscape (conservation of core habitats and corridors, and reducing species mortality on the roads) must be considered by wildlife managers in Iran.

## Introduction

Human activities have threatened large carnivores through habitat loss, fragmentation and isolation at multiple scales^1–3^. With increasing loss and fragmentation of habitats, there is a crucial need to identify the most important areas for conservation actions^4^. The conservation areas (CAs) network should protect both core habitats and corridors^5^. However, several studies report that the existing CAs are primarily small and insufficient to support large carnivores with extensive home range and low population density^6–9^.

Large carnivores are considered keystone species because they are apex predators and they are umbrella species. Thus conserving carnivores helps regulate prey species and leads to sympatric biota’s conservation^10^. Large carnivores are also indicator species because of their sensitivity to habitat fragmentation^11^. Therefore, large carnivores are often selected by researchers as the surrogate species^12^. However, it is often difficult to identify core habitats and corridors of large carnivores due to their mainly cryptic and nocturnal nature^13,14^. Species distribution models (SDMs)^15^ have come to aid researchers in predicting suitable habitats of large carnivores. In addition, SDMs were applied as input data to predict movement corridors used for dispersal and gene flow among core habitats in order to direct management of the species^16–18^. Identified corridors can be used to direct land managers, for example, managers can prioritize improving wildlife road crossings in areas when roads cross corridors^19–21^.

Roads have adverse effects on wildlife populations, including large carnivores, and particularly threatened species^22–25^. Roads fragment continuous habitats and facilitate human access to pristine natural areas^26^. Furthermore, anthropogenic caused mortalities, road collisions being one, are the main concerns for the conservation of threatened large carnivores^24^. Several studies have been done in Iran that tested relationship between road collisions of large carnivores and ecological corridors^9,19,23^; but none focused on the striped hyaena (*Hyaena hyaena* Linnaeus, 1758).

The striped hyaena occurs in Asia from the Indian subcontinent to the Levant (including 20 countries) and most parts of Africa except the southern part (including 18 countries)^27,28^. According to the IUCN Red List, the striped hyaena has been categorized as a near threatened species (NT), because of persecution (mainly poisoning the carrions) and decreasing domestic and natural carrions^29^. The reason for this decrease is the reduction in other sympatric large carnivores’ populations and their prey^29,30^. The striped hyaena is a classic omnivorous scavenger^30,31^, which scavenges a variety of foods, including vertebrates, insects and other invertebrates, dried bones, fruits, human organic waste, etc.^32,33^. Striped hyaenas are one of the least studied large carnivores in Iran and there is limited data available on their habitat needs and spatial distribution. In Iran, the striped hyaena has a widespread distribution; however, its population has decreased severely^27^. The main causes are habitat loss and anthropogenic activities such as conversion of the natural grasslands to agricultural lands, poaching, poisoning the carrions, using organs for medicine and superstitious beliefs, and road collisions^27,34,35^. For this regard, identifying striped hyaena habitat suitability, core habitats and connectivity among them are prerequisite steps to delineate management strategies aiming at human-striped hyaena co-existence. This species can be considered as a surrogate species and identifying core habitats and connectivity network can help in locating new CAs and protecting other co-existence species.

This study was carried out in order to (1) assess the habitat suitability of the striped hyaena to predict the core habitats and corridors in Khuzestan area, southwestern Iran, (2) compare identified core habitats and corridors with existing CAs, and (3) overlay the road collisions of the striped hyaena with the predicted corridors.

## Materials and methods

### Study area

**Figure 1:**
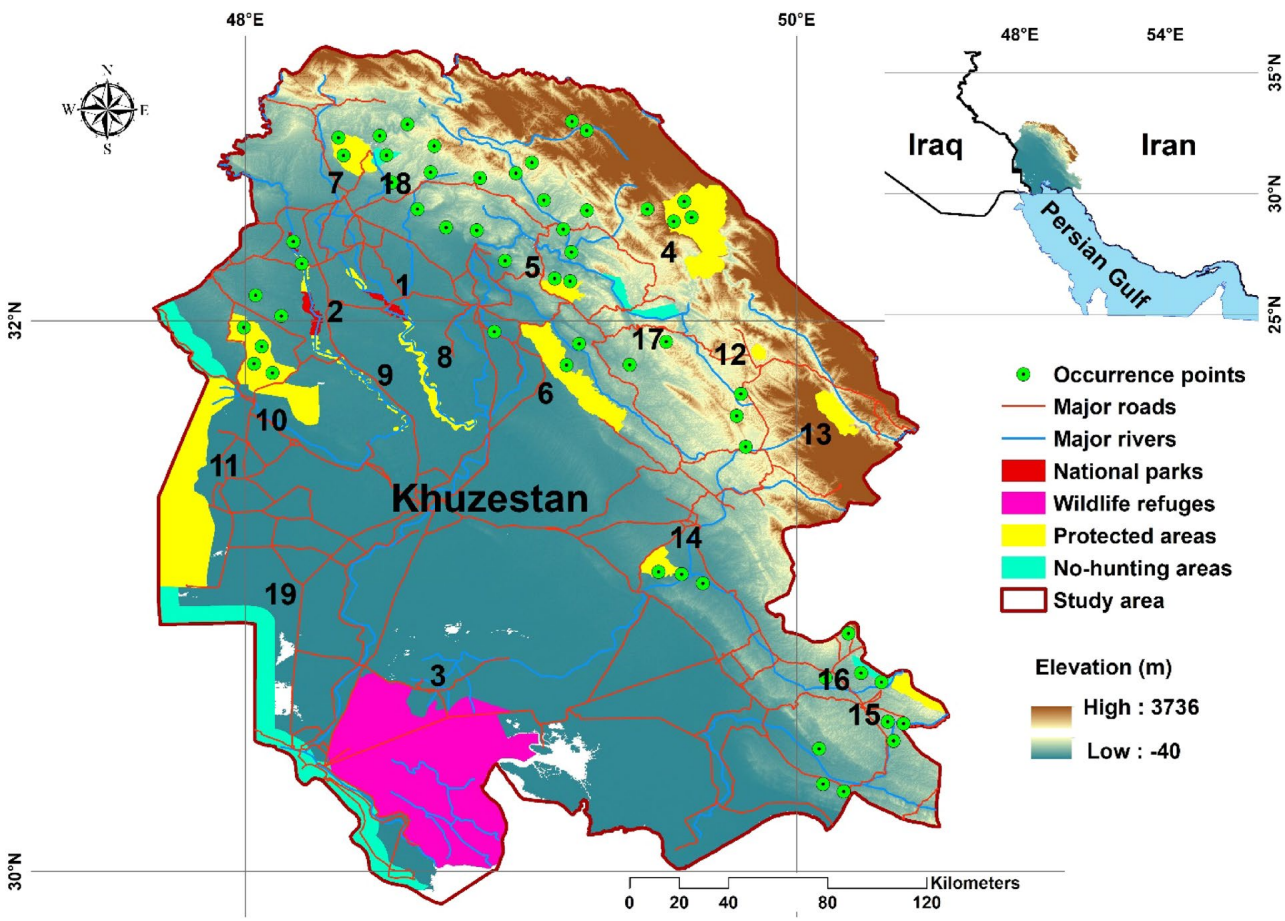
Study area including Khuzestan area in southwest of Iran, occurrence points and conservation areas (names of conservation areas are available in Table S1). ArcGIS software version 10.1 (https://www.esri.com/en-us/arcgis/products/arcgis-pro/resources) was used to generate the figure.

Khuzestan area is a province located the southwest of Iran (area: 64,057 km^2^) (Fig. 1). Northeast of the study area includes mountainous areas with cold winters (mean 6 °C) and mild summers (mean 25 °C) with the dominant plant species of *Hordeum marinum* Huds., *Onosma rosellatum* Lehm. and *Ducrosia anethifolia* (DC.) Boiss. Other parts of the study area include vast arid plains with mild winters (mean 17 °C) and hot summers (mean 37 °C)^36^ with dominant plant species of *Onopordum heteracanthum* C.A.Mey., *Chrozophora hierosolymitana* Spreng. and *Capparis spinosa* L. CAs covers about 13% of the study area; includes two national parks (NPs), one wildlife refuge (WR), 12 protected areas (PAs) and four no-hunting areas (NHAs) (Supplementary Information: Table S1, Fig. 1). NPs, WRs and PAs have the highest conservation priorities in Iran, respectively and NHAs were established for poaching control and have the lowest conservation priorities^37^. NPs, WRs, PAs and NHAs are near to the II, III, IV and IV-VI of the IUCN categories, respectively^37^. The density of major roads is 70.8 m/km^2^ in the study area. The study area includes several long rivers (e.g., Karoon, Karkherh and Dez) with a density of 40 m/km^2^ (Fig. 1). Brown bear (*Ursus arctos*), Persian leopard (*Panthera pardus saxicolor*), striped hyaena, grey wolf (*Canis lupus*), golden jackal (*Canis aureus*), caracal (*Caracal caracal*), jungle cat (*Felis chaus*), wild cat (*Felis lybica*) and honey badger (*Mellivora capensis*) are the main carnivores in the study area^38,39^.

### Occurrence points̕ collection and environmental variables

Occurrence points of the striped hyaena in the study area were collected by Khuzestan provincial office of the Department of Environment (DoE) guards and experts, including the third author, during 2015–2020. A number of 58 occurrence points were obtained for the striped hyaena in the study area. Spatial-autocorrelation was reduced by using the radius of 4 km around each occurrence point according to mean maximum distance moved (MMDM) by the striped hyaena in arid areas of India^40^ using the Spatially Rarify Occurrence Data tool in the SDMtoolbox^41^. Only one occurrence point was excluded and 57 occurrence points were used for habitat modeling of the striped hyaena in the study area (Table S2).

All related environmental variables i.e. topographic, climatic, land cover, safety and protection, water resources and human disturbance variables, were considered for habitat modeling of the striped hyaena in the study area (Table S3). Digital Elevation Model (DEM) was download from http://srtm.csi.cgiar.org as the elevation variable with a resolution of 250 m. This data was derived from the 90 m Shuttle Radar Topography Mission (SRTM, http://earthexplorer.usgs.gov). DEM was used to calculate the slope (using Surface Tool) and the topographic roughness index (standard deviation of elevation value of DEM᾽s cells within the radius of 4 km) using ArcGIS software version 10.1^42^ (https://www.esri.com/en-us/arcgis/products/arcgis-pro/resources). A categorical climatic layer created based on De Martonne's classification with eight classes (from very humid to very arid) was used for habitat modeling of the striped hyaena in the study area.

Forests, grasslands and agricultural lands cover-types were derived from the land-cover map of Iran. A circle-moving window with a 4 km radius was used to create density maps of these three cover-types. Normalized Difference Vegetation Index (NDVI) was created by the 16-day composite MODIS data (MODIS MYD 13Q1 V6 map at 250 m cell size; http://earthexplorer.usgs.gov) according to the mean values of the year 2020. Because CAs protect animals from hunting or other human disturbances, distance to CAs was considered. We considered distance to rivers given the importance of water resources for carnivores^13^ and because the striped hyaena is found in the areas, where water is available within 10 kilometers^40,43^. Distance to roads was assessed as a predictor. Furthermore, another human disturbance variable, distance to villages was considered because villages attract striped hyaenas to scavenge dead domestic and organic wastes^30^.

To reduction and choose the optimal variables for habitat modeling of the striped hyaena, the MaxentVariableSelection package^44^ in R version 3.6.0^45^ (https://www.r-project.org/) was employed by setting a contribution threshold of 1%, regularization multiplier of 1 to 5 with increments of 0.5 and inter-correlation of 0.7. Eight variables with the highest area under the curve (AUC) of receiver operating characteristic (ROC) and the lowest Akaike information criterion (AIC) were chosen by package (Table 1). Variance Inflation Factor (VIF) of selected variables in step 1 was checked using the usdm package^46^ in R to exclude variables with VIF > 3 (threshold suggested by Zuur et al.^47^). None of the selected variables was excluded due to low VIFs (Table 1).

**Table 1:** Environmental variables using for habitat modeling of the striped hyaena in the study area.

Variables category	Variables	Selected by MaxentVariableSelection	VIF
Topography	Elevation	Selected	1.65
	Slope	–	–
	Roughness	–	–
Climate	Categorical climate	Selected	1.13
Land-cover	Forests density	–	–
	Grasslands density	–	1.29
	Agricultural lands density	Selected	1.69
	NDVI	Selected	1.25
Prey availability	Distance to CAs	Selected	1.37
Water resources	Distance to rivers	Selected	1.2
Human disturbance	Distance to roads	Selected	1.5
	Distance to villages	Selected	1.18

### Habitat modeling

Habitat suitability prediction of the striped hyaena was carried out using an R-package biomod2^48^ as an ensemble modeling approach. The predictive accuracy of the habitat suitability model improves by combining different suitability models^49,50^. Four regression-based models, five machine-learning models and one profile model were implemented for the primary habitat modeling in Biomod2 (Table 2), and four models with AUC > 0.9 and True Statistic Skill (TSS) > 0.75 thresholds were chosen as the best fit^51^. According to method used by Kaboodvandpour et al.^4^, six hundred pseudo-absence points were randomly created across the study area (separated by > 4 km from each other) and outside of the 4 km radius circle around each occurrence point. Totally, 658 points (600 pseudo-absence points + 58 occurrence points) and eight environmental variables were used for habitat modeling of the striped hyaena in the study area by using four models of GLM, MaxEnt, GBM and RF (Table 2). Then, map of ensemble habitat suitability was created in biomod2 by weighted-average of models values^48^. The mean of variables̕ contribution of related models was calculated in Biomod2. In addition, response curves of occurrence points to the variables for the two most accurate models were illustrated in the study area. According to the method of Wan et al.^52^, the ensemble suitability map was converted into a resistance map. The linear method in rescale by function tool in ArcGIS software and the negative exponential function (R = 1000^(−1 × Habitat Suitability)^)^53^ were used to create the resistance map in the range of 1 (lowest resistance) to 10 (highest resistance)^52^.

**Table 2:** Different prediction models used for habitat modeling of the striped hyaena in the study area.

Prediction model category	Prediction model	AUC	TSS
Regression-based models	Generalized linear model (GLM)*	0.914	0.794
	Generalized additive model (GAM)	0.76	0.678
	Multivariate adaptive regression splines (MARS)	0.845	0.69
	Flexible discriminant analysis (FDA)	0.87	0.643
Machine-learning models	Maximum entropy (MaxEnt)*	0.93	0.816
	Generalized boosting model (GBM)*	0.943	0.822
	Random forest (RF)*	0.921	0.835
	Classification tree analysis (CTA)	0.755	0.569
	Artificial neural network (ANN)	0.839	0.572
Profile model	Surface range envelop (SRE)	0.764	0.548

*Selected for final habitat modeling.

### Core habitats and corridor modeling

Corridor modeling was carried out by using Universal Corridor (UNICOR) software version 1.0^54^ (https://www.fs.usda.gov/treesearch/pubs/40686). The advantage of this software was a dispersal threshold defined by the user to predict core habitats by using resistant kernel^9^. Connectivity prediction created factorial least-cost path routes with the highest probability of dispersal^54,55^.

According to the studies of Kruuk^33^ and Wagner^31^, the distance threshold of 70,000 (movement abilities of 70 km) was used in resistant kernel analyses. The resistance map was used to identify core habitats of the striped hyaena with the selected scenario. The buffered least-cost paths were then combined through summation to produce the corridor map between all pairs of occurrence points^55^. The contiguous map of core habitats was converted to a categorical map based on > 10% of the highest records for the species^8,37^. In other words, contiguous areas with resistance values less than 10 (0–100) were chosen as core habitats. This work was carried out for corridors as well. Only the categorical corridors out of core habitats were considered. The densities of roads and rivers were calculated for each core habitats and corridors. The coverage of CAs with core habitats and categorical corridors of the striped hyaena was calculated separately in the study area.

### Contribution of core habitats for connectivity

The Conefor software version 2.6^56^ (http://www.conefor.org/coneforsensinode.html) was used to measure dPC (i) as the reduction of landscape connectivity associated with the loss of core i^57^, and three subsections of dPCintra, dPCflux and dPCconnector^57^. dPCintra (i) measures the contribution of core i to landscape connectivity associated with its area and suitability while dPCflux measures the contribution of core i to landscape connectivity associated with dispersal between it and other core areas on the landscape. dPCconnector measures the contribution of core i to landscape connectivity due to its role as a stepping stone, connecting other core areas to each other^58,59^. To prepare the data for Conefor (i.e., node and distance files), categorical core habitats of the striped hyaena were applied in Conefor Input ArcGIS extension (http://www.jennessent.com/arcgis/conefor_inputs.htm).

### Road collisions and predicted corridors

The procedure of spatial randomization test was done to evaluate the occurrence of striped hyaena road crossing within the predicted corridors^60,61^. A number of 10,000 random points were created along the dangerous roads (roads with record of vehicle collisions, 536 km) in the study area, whereas 30 road observations including 18 successful crossing and 12 collisions records were documented by DoE guards and experts, including the third author with random patrol monitoring during 2015–2020. These 30 road observations were not included in the set of observations used to fit the habitat model. The median value of resistant kernel (predicted connectivity) of road observations was compared with median values of 10,000 random points using a non-parametric test with 10^7^ iterations of 30 locations.

## Results

### Habitat modeling and variables contribution

**Figure 2:**
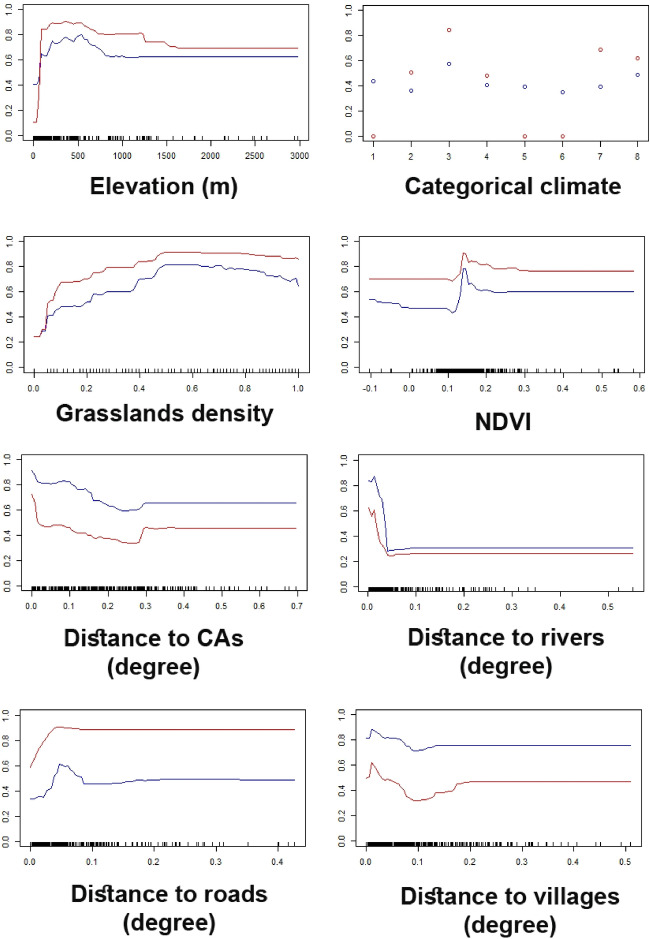
Response curves of occurrence points of the striped hyaena to the environmental variables (the two most accurate models of RF [red] and GBM [blue] were considered). Y-axis represents the probability of the striped hyaena occurrence. X-axis of categorical climate variable represents: (1) sea and lake, (2) semi-humid, (3) semi-arid, (4) humid, (5) very arid, (6) very humid, (7) Arid and (8) Mediterranean (each 0.1 geographical degree in the study area is approximately equal to 13.2 km).

**Figure 3:**
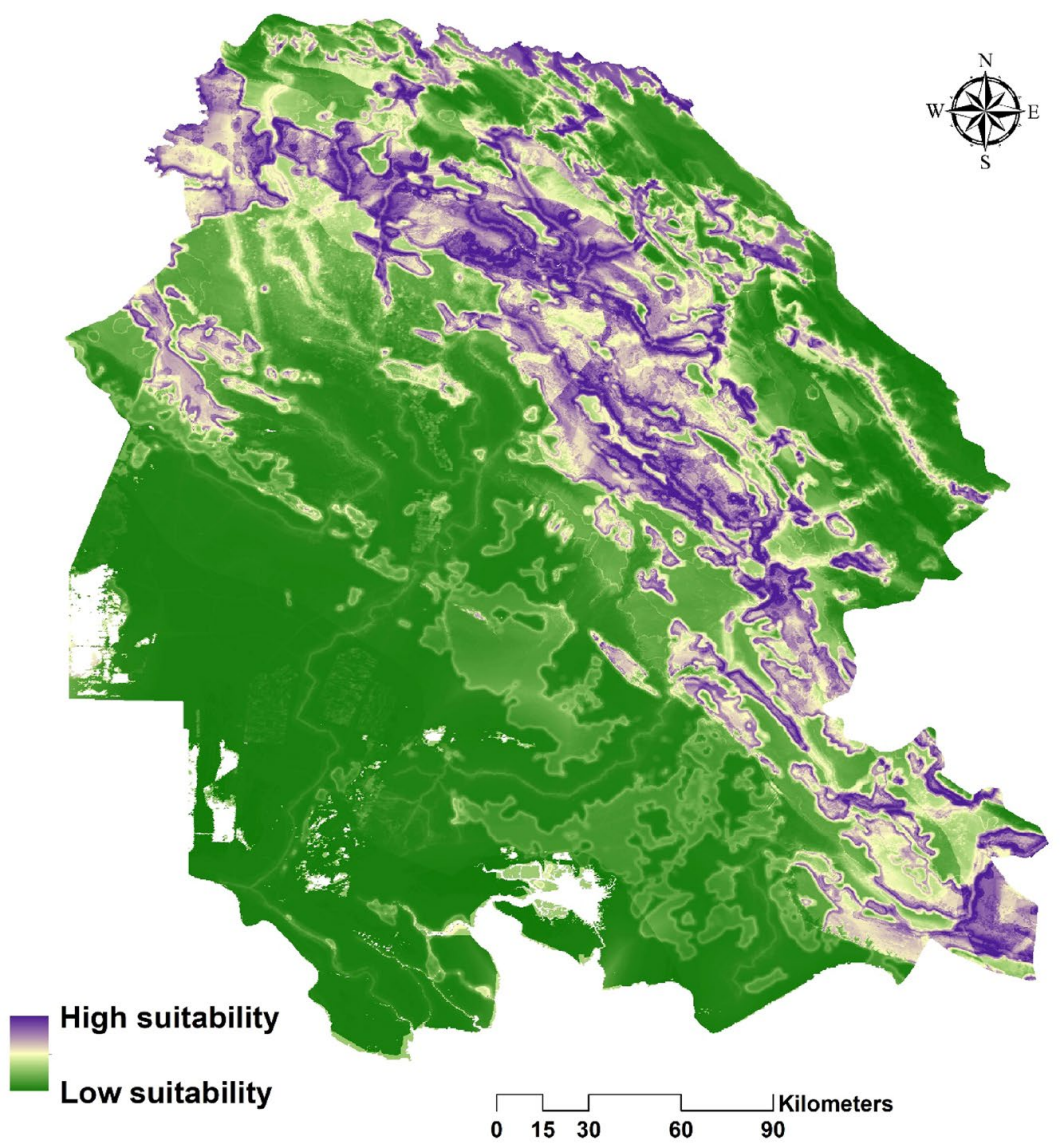
Ensemble habitat suitability map for the striped hyaena in the study area based on the four optimal models of GLM, MaxEnt, RF, and GBM. ArcGIS software version 10.1 (https://www.esri.com/en-us/arcgis/products/arcgis-pro/resources) was used to generate the figure.

Habitat suitability prediction revealed that elevation, distance to CAs, categorical climate and grasslands density were the most influential variables for predicting the occurrence of the striped hyaena in the study area (Table S4). The optimal range of elevation for the striped hyaena occurrence was 500–1500 m in the study area, and stabilized at 2000 m. The striped hyaena occurred mainly in semi-arid, arid and Mediterranean areas, respectively. As NDVI in natural grasslands increased, the probability of the striped hyaena occurrence increased and then stabilized at 0.2 (from − 1 to 1). As distance to CAs and distance to rivers increased, the probability of striped hyaena occurrence decreased. By increasing distance to roads, the probability of the striped hyaena occurrence increased gradually and then stabilized at about 13 km (Fig. 2). Finally, probability of striped hyaena occurrence increased with increasing distance to villages and then stabilized at about 26 km. Ensemble suitability map showed that hills and hillsides of northeast, east and southeast of the study area had the highest suitability for the striped hyaena (Fig. 3). Habitat suitability models of GLM, MaxEnt, GBM and RF are shown at Supplementary Information (Figure S1).

### Core habitats and corridors

**Figure 4:**
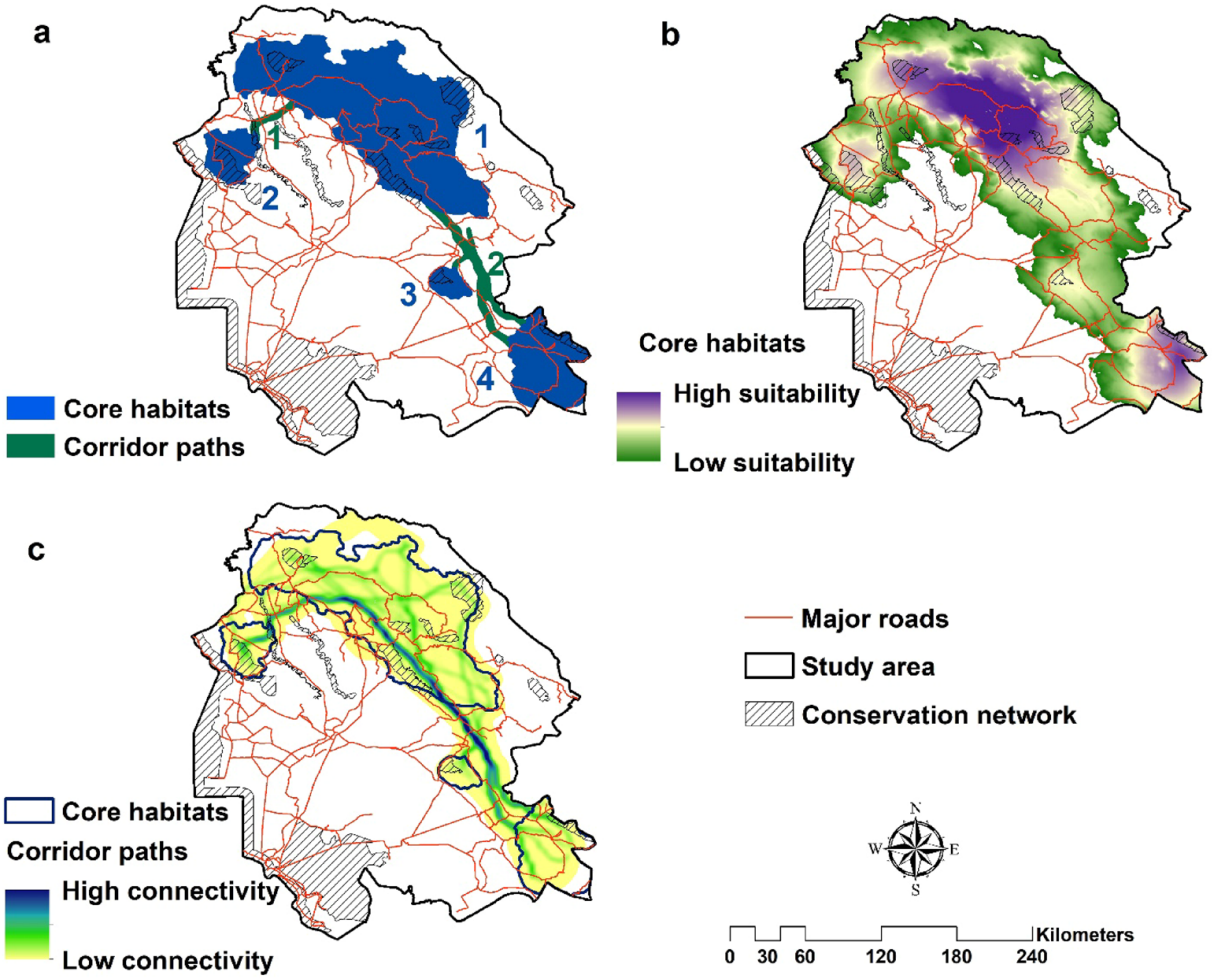
Core habitat and corridors for the striped hyaena in the study area (a: Categorical core habitats and corridor paths, b: Contiguous core habitats and c: Contiguous corridor paths). ArcGIS software version 10.1 (https://www.esri.com/en-us/arcgis/products/arcgis-pro/resources) and UNICOR software version 1.0 (https://www.fs.usda.gov/treesearch/pubs/ 40,686) were used to generate the figure.

Four core habitats were identified, covering 25% of the study area (Fig. 4; Table 3). The largest habitat patch was Core1, located northeast of the study area (about 11,400 km^2^) (Fig. 4). The second-largest habitat patch was Core4, located southeast of the study area (about 2700 km^2^) (Fig. 4, Table 3). One NP, eight PAs and three NHAs were located within identified core habitats. About 11% of the predicted core habitats were covered by CAs (Table 3). Core2 had the highest percentage of coverage with CAs (33%). Core2 and Core3 had the highest density of roads (81.1 m/km^2^) and rivers (83.95 m/km^2^), respectively (Table 3).

**Table 3:** Properties of predicted core habitats and corridors for the striped hyaena in the study area (number of core habitats and corridors are available at Fig. 4).

	Number	Area (km^2^)	Protected		Road density (m/km^2^)	River density (m/km^2^)	Number of CAs inside cores and corridors			
			Area (km^2^)	%			NP	WR	PA	NHA
Core habitats	1	11,392	1129.69	9.92	64.5	59.08	–	–	4	2
	2	1195.4	394.74	33.02	81.1	12.39	1	–	2	–
	3	512.89	98.06	19.12	50	83.95	–	–	1	–
	4	2726.07	140.61	5.16	65.56	55.64	–	–	1	1
	Total	15,826.36	1763.1	11.14	69.16	55.87	1	–	8	3
Corridors	1	209.29	16.62	7.94	231.97	102.84	1	–	–	–
	2	1043.41	–	–	81.52	71.58	–	–	–	–
	Total	1253.67	16.62	1.33	106.55	76.79	1	–	–	–

The connectivity for the striped hyaena in the study area was maintained between core habitats from northwest to southeast (Fig. 4). Two main corridors were detected among core habitats. Corridor1 had moderate connectivity between Core1 and Core2 (Fig. 4). Corridor2 among Core1, Core3 and Core4 had high connectivity between the northeast and southeast of the study area. This corridor had two branches: one from Core1 to Core3 and another from Core1 to Core4. Only one NP was located within corridors. Overall, less than 2% of corridors were covered by CAs (Table 3) and in Corridor2 was outside of a CA. Corridor1 had the highest density of roads (231.97 m/km^2^) and rivers (102.84 m/km^2^) (Table 3).

### Contribution of core habitats for connectivity

Based on dPC index at the estimated dispersal distance scenario, Core1 had the highest contribution to habitat connectivity (Table 4). Based on the results of dPCintra and dPCflux, Core1 had the highest intrapatch connectivity and the highest flux according to patch area and the position within the landscape. Core4 had the highest second contribution. Core1 had the highest contribution as the stepping-stone (Table 4).

**Table 4:** Values of dPC index and its three fractions (intra, flux and connector) calculated for predicted four core habitats at the dispersal scenario of 70 km (number of core habitats are available in Fig. 4).

	dPC	dPCintra	dPCflux	dPCconnector
Core1	93.87	64.01	29.02	0.84
Core2	13.06	0.7	12.36	0
Core3	5.29	0.13	5.16	0
Core4	20.11	3.67	16.44	0

### Road collisions and predicted corridors

**Figure 5:**
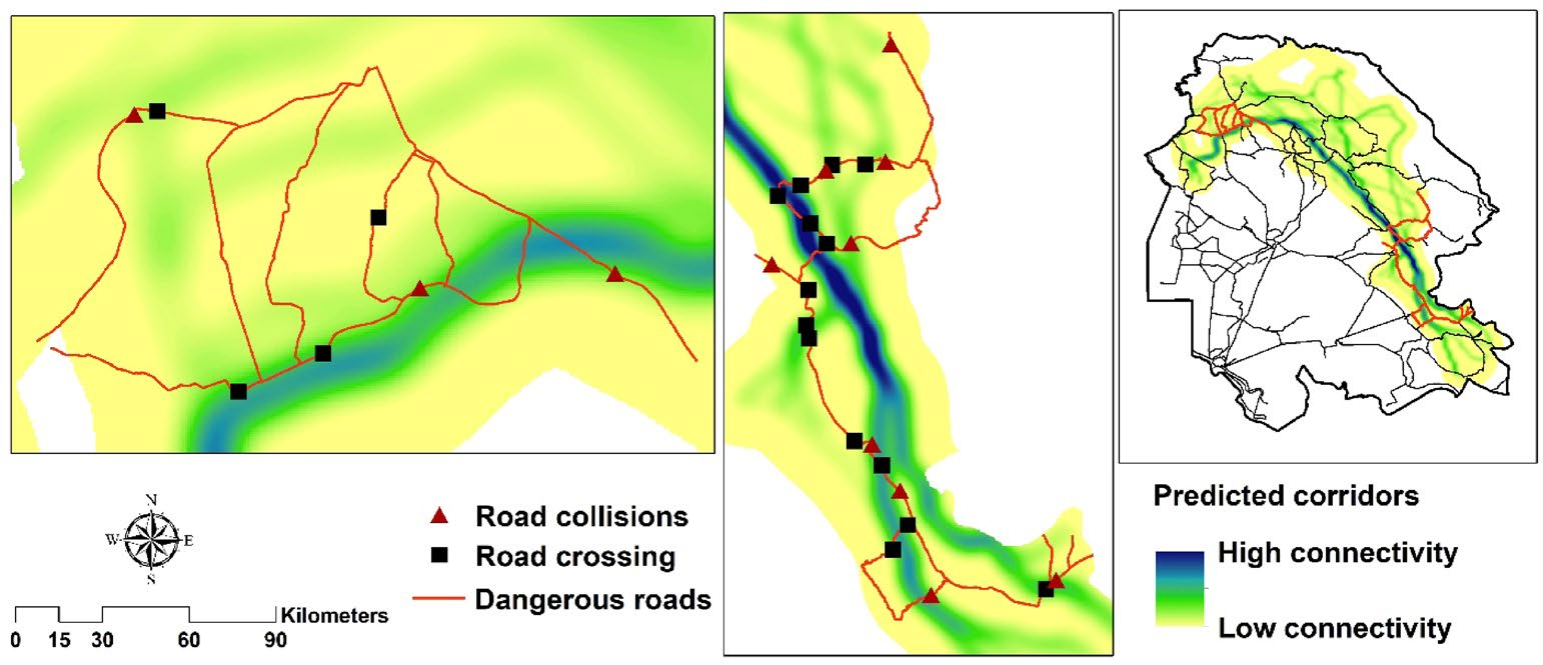
Predicted corridors, striped hyaena road observations (18 crossing + 12 collisions, data collected during 2015–2020) and roads in the study area. ArcGIS software version 10.1 (https://www.esri.com/en-us/arcgis/products/arcgis-pro/resources) and UNICOR software version 1.0 (https://www.fs.usda.gov/treesearch/pubs/40686) were used to generate the figure.

**Figure 6:**
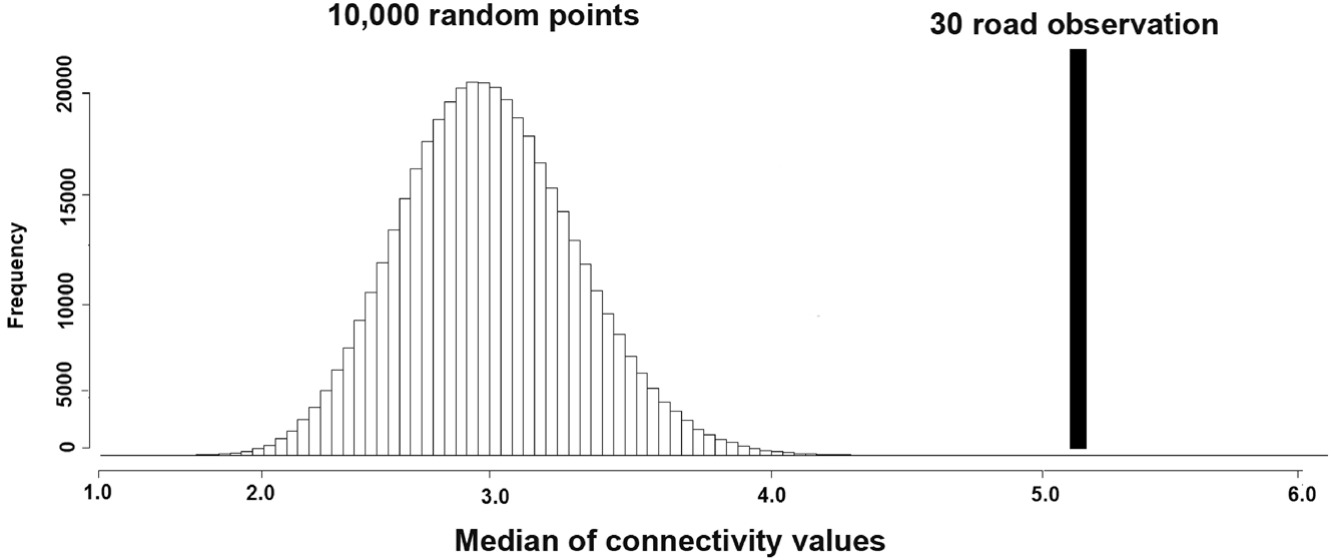
Spatial randomization test: 30 road observations (18 crossing + 12 collisions) of the striped hyaena and 10,000 random points along the dangerous roads of the study area.

Out of 12 road collisions for the striped hyaena, four were males, seven were females and one was a cub. Six road collisions occurred during winter (from January to March), four during spring (from April to June) and two during summer (from July to September) (Table S5). Predicted corridors, crossed by roads represented a high risk for striped hyaenas (Fig. 5). The spatial randomization test revealed that observations points (crossing + collisions) were more likely to be within corridors than random points (Fig. 6). Observation points had a significantly higher connectivity score than the randomly selected locations (*P* < 0.001).

## Discussion

We found four variables of elevation, distance to CAs, categorical climate and grasslands density to be significant predictors of striped hyaena occurrence in Khuzestan area, southwestern Iran. We identified four core habitats and two corridors that have the potential to maintain connectivity. The largest core habitat (Core 1) had the highest priority for conservation. Only about one tenth of core habitats was protected by CAs.

Rieger^43^ reported that the striped hyaena occurred in Iran at elevations up to 2250 m. Here, we predicted slightly lower value of 2000 m. Our habitat modeling predicted that striped hyaenas are limited by higher elevation, however, that is disagreed with the results of Shamoon and Idan^62^. In our study area, the striped hyaena preferred mainly semi-arid and arid areas with a moderate density of grasslands, and this finding is supported by Leakey et al.^63^.

The striped hyaena were more likely to occur near rivers, and this finding was supported by Rieger^43^, and Singh et al.^40^, and near the villages, which was mentioned earlier by Singh et al.^40^, Akay et al.^28^ and Farhadinia et al.^64^. In addition, Bhandari et al.^65^ found that the striped hyaena prefers open landscapes along rivers and human settlements, because of suitable cover and access to resources. Other studies found presence of domestic animals in the striped hyaena scats, which was indicative of frequent near human settlements^30^. In Iran, preying upon livestock by the striped hyaena is rare, and this species approaches the villages for feeding on carrions of domestic animals^64^.

### Core habitats, corridors and CAs

We identified four core habitats for striped hyaenas; however, all had about 10% protection status. Core1 is the largest patch of suitable habitat and occupies a central location relative to other habitat in the study area, which has made the core habitat with the highest flux and as a connector (stepping-stone) within the landscape. Therefore, Core1 had the highest contribution for connectivity in the study area. Only about 12% of core habitats were protected by CAs, which was less than the amount set for near threatened and threatened mammals in Iran^66^ (i.e., 20%). In addition, Farashi et al.^67^ reported 66% coverage of CAs with suitable areas of the striped hyaena in Iran, which is remarkably higher compared to the obtain value in this study (11.4%). That is why in their study, occurrence points (centroid of the area of occupancy) of the Atlas of Mammals of Iran^39^ were used for habitat modeling of the striped hyaena with insufficient occurrence points (just one point) in Khuzestan area (our study area). In this study, by applying sufficient occurrence points for habitat modeling of the striped hyaena, predicted large core habitats in this area needs more CAs to cover unprotected core habitats. Furthermore, establishing more strictly conservation areas is politically challenging. Therefore, we strongly recommend establishing new less strictly conservation areas, such as NHAs. In addition, a small proportion of corridors was protected by CAs. This means that more CAs are needed for conservation of corridors of the striped hyaena in the study area.

### Road collisions and predicted corridors

Road collisions were mainly observed in the predicted corridors and a few in the edge of core habitats. However, crossings and collisions occurred even in areas of low predicted connectivity. Corridor1 had a relatively high density of roads and it was bisected about 10 times by roads. Consequently, some of road collisions observed here. In return, Corridor2 had lower density of roads and was bisected similar times with Corridor1. However, a higher number of road collisions were observed here, because higher connectivity caused more individuals movement of striped hyaenas in Corridor2 (from Core1 to Core3 and Core4 and vice versa). Areas that were predicted to be corridors had more road kill observations and that fits out hypothesis that striped hyaenas will be in greater risk of road collisions when moving between core habitats. Our results support previous findings on the use of resistant kernel and factorial least-cost path analyses for effective prioritization of dangerous roads^23,61^.

### Conservation implications for the striped hyaena

With increasing human population and habitat loss, the pressure on the large carnivores, including the striped hyaena has increased^2^. Large core habitats could help the striped hyaena meet its ecological requirements^11^. Increasing the amount of CAs is necessary for the conservation of the large carnivores^4^, and in particularly for the striped hyaena as demonstrated in this study. In addition, maintaining landscape connectivity is necessary for carnivores^68^, consequently, population gene diversity is conserved^16^. We urge decision makers to take into account the results of this study when planning corridors between core habitats.

Striped hyaenas movements between core habitats may result in more human-hyaena interactions and therefore additional mitigation efforts is necessary to ensure the safety of the species. For example, increasing local knowledge about the behavior of the striped hyaena (feeding on domestic carrions) and low probability of attacks on domestic animals could be effective for conservation of the species^64,69^. Facilitating safe wildlife crossing of roads e.g. use of multiple warning signs in dangerous roads in high risk road sections could mitigate the number of road collisions^19^. Actually, adaptive management plan throughout the landscape (conservation of core habitats and corridors, and reducing species mortality on the roads) must be considered by DoE managers^23^.

## Conclusions

This study was carried out in Khuzestan area in southwestern Iran (mainly arid and semi-arid areas). Four core habitats were detected in this study. The largest one is located in the northeast of the study area. The connectivity was maintained from northwest to southeast of the study area with two main corridors. The result of this study can help direct future conservation plans for the striped hyaena.

## Supplementary Information

Supplementary Information.
