# Supplementary material for: Identifying core habitats and corridors of a near threatened carnivore, striped hyaena (Hyaena hyaena) in southwestern Iran
Source: Sci Rep. 2022 Mar 2;12:3425. doi: 10.1038/s41598-022-07386-y (PMC8891386; doi:10.1038/s41598-022-07386-y)
Supplement: Supplementary file 1 — Supplementary Information. [file 41598_2022_7386_MOESM1_ESM.doc]

**Supplementary Information**

**Figures**

**
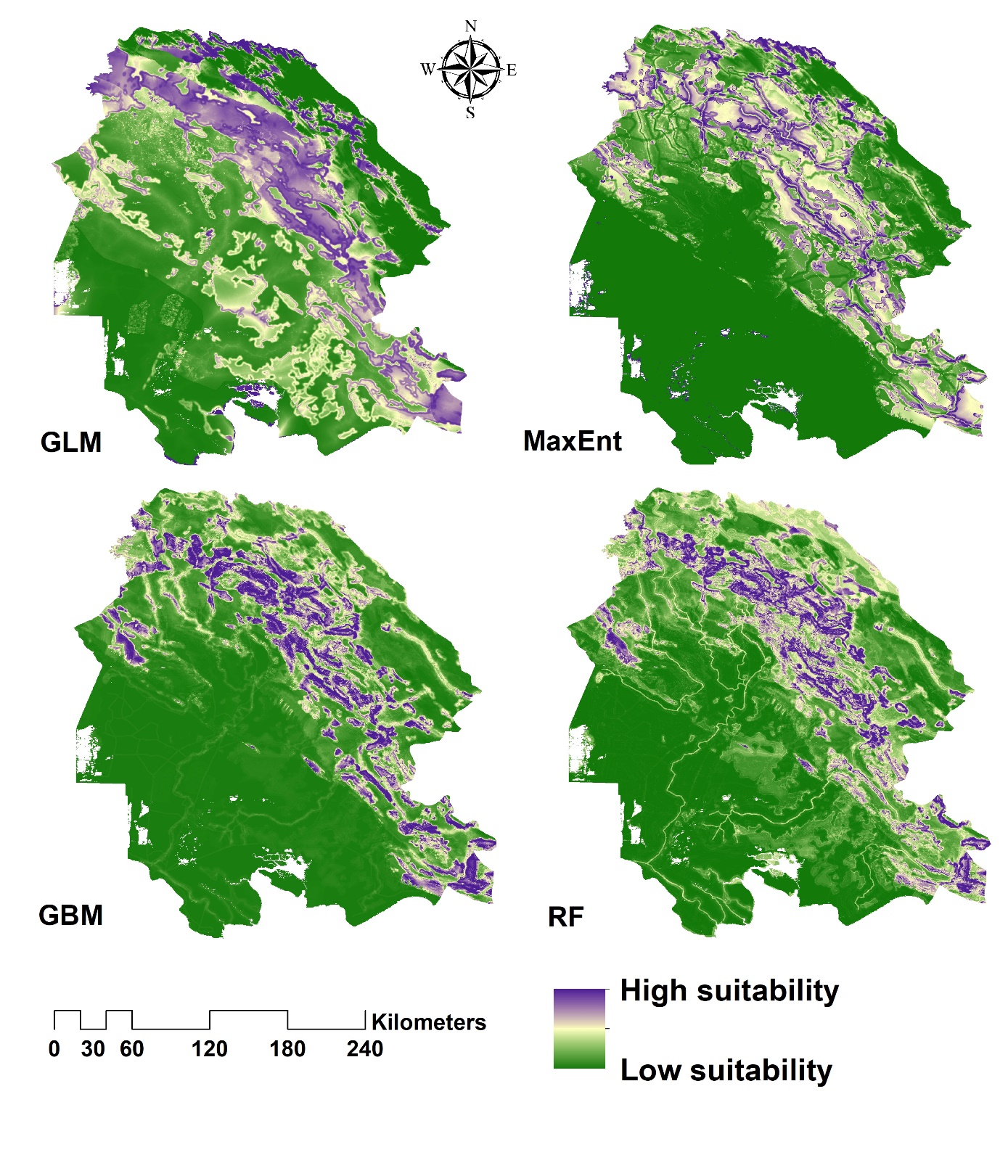
**

**Figure S1.** Optimal four habitat suitability models for the striped hyaena in the study area. ArcGIS software version 10.1 (https://www.esri.com/en-us/arcgis/products/arcgis-pro/resources) was used to generate the figure.

**Tables**

**Table S1.** List of Conservation areas including national parks, wildlife refuges, protected areas and no-hunting areas in the study area

| Conservation areas category | Name | Area (km^2^) | Number in Figure 1 |
| --- | --- | --- | --- |
| National parks  (NPs) | Dez NP | 74.77 | 1 |
|  | Karkheh NP | 41.18 | 2 |
| Wildlife refuge (WR) | Shadegan WR | 3289.26 | 3 |
| Protected areas  (PAs) | Shimbar PA | 540.21 | 4 |
|  | Haft-Shahidan PA | 93.78 | 5 |
|  | Koraee PA | 406.44 | 6 |
|  | Chehelpa PA | 169.88 | 7 |
|  | Dez PA | 178.95 | 8 |
|  | Karkheh PA | 83.52 | 9 |
|  | Mishdagh PA | 541.8 | 10 |
|  | Hoorolazim PA | 1175.81 | 11 |
|  | Miangaran PA | 24.76 | 12 |
|  | Shaloo and Mongasht PA | 125.55 | 13 |
|  | Dymeh PA | 98.12 | 14 |
|  | Khiz and Sorkh PA | 98.96 | 15 |
| No-hunting areas  (NHAs) | Badil NHA | 51.54 | 16 |
|  | Shou and Lander PA | 125.5 | 17 |
|  | Ghaleh Shadab PA | 40.87 | 18 |
|  | The width of seven-kilometers on the border | 1523.28 | 19 |

**Table S2.** Sources of occurrence points of the striped hayaena in the study area.

| Sources of occurrence points | Number of occurrence points in the study area |
| --- | --- |
| Direct observation* | 23 |
| Camera traps+ | 21 |
| Den sites | 7 |
| Dead body (because of diseases, gun shooting or poisoning) | 6 |

*During 2015-2020 (six years), different parts of the study area were surveyed randomly by guards and experts, including the third author to detect carnivores’ occurrence e.g. the striped hyaena. About 1400 efforts were carried out in the study area during the mentioned time, which led to 23 direct observations, 7 den sites and finding 6 dead bodies (except road collisions). The guards have received necessary training and technics to identify various carnivores including the striped hyaena.

**+** Camera traps were deployed to detect various carnivores for about 35 days in 42 stations with a total effort of 1470 trap nights in areas with more probability of carnivores’ occurrence by using the dominant type of Cuddeback Capture (Green Bay, WI, USA) model. Camera traps were placed approximately 40 cm up the ground on natural features. The number of 21 camera traps in north and northeast parts of the study area captured the striped hyena (43 captured).

**Table S3**. All related environmental variables and their sources for habitat modeling of the striped hyaena in the study area

| Category | Variables | Source |
| --- | --- | --- |
| Topographic variables | Elevation (DEM) | [http://srtm.csi.cgiar.org](http://srtm.csi.cgiar.org/)  Jarvis et al., 2008 |
|  | Slope | Elevation |
|  | Topographic roughness index |  |
| Bioclimatic variable | Categorical climatic layer | IRIMO, 2017 |
| Land-cover | Forests density | FRWMO, 2010 |
|  | Grasslands density |  |
|  | Agricultural lands density |  |
|  | Normalized Difference Vegetation Index (NDVI) | [http://earthexplorer.usgs.gov](http://earthexplorer.usgs.gov/) |
| Safety and protection | Distance to conservation areas (CAs) | DoE, 2018 |
| Water resources | Distance to rivers |  |
|  | Distance to wetlands |  |
| Human disturbance | Distance to villages |  |
|  | Distance to roads |  |

**Table S4.** Variables contribution (mean and standard deviation) in the habitat modeling of the striped hyaena in the study area.

|  | Elevation | Categorical climate | Grasslands density | NDVI | Distance to CAs | Distance to rivers | Distance to roads | Distance to villages |
| --- | --- | --- | --- | --- | --- | --- | --- | --- |
| Mean | 20.3 | 15.7 | 13.4 | 6 | 18.1 | 10.2 | 6.8 | 9.5 |
| SD | 2.9 | 2.2 | 2.3 | 1.3 | 2.4 | 1.4 | 2.9 | 1.9 |

**Table S5**. Road collisions of the striped hyaena in the study area during 2015-2020

| Number of striped hyaena | Sex | Season | Year |
| --- | --- | --- | --- |
| 1 | Male | Summer | 2015 |
| 1 | Male | Spring | 2016 |
| 1 | Female | Winter | 2016 |
| 1 | Female | Spring | 2017 |
| 1 | Female | Spring | 2018 |
| 1 | Female | Winter | 2018 |
| 1 | Male | Summer | 2019 |
| 1 | Female | Winter | 2019 |
| 1 | Male | Spring | 2019 |
| 1 | Female | Winter | 2020 |
| 2 | Female with its cub | Winter | 2020 |

**References**

DoE (Department of the Environment of Iran). Department of the Environment of Iran. https://www.doe.ir. Accessed 1 April 2020, (2018).

FRWMO (Forest, Range and Watershed Management Organization of Iran). Iranian forests, range and watershed management organization national land use/land cover map, (2010).

IRIMO (Islamic Republic of Iran Meteorological Organization). Climate data-base, Iranian cities, from 1993 to 2017. [https://www](https://www/).irimo.ir. Accessed 1 October 2020, (2017).

Jarvis, A., Reuter, H. I. Nelson, A. & Guevara, E. Hole-filled seamless SRTM data V4, International Centre for Tropical Agriculture (CIAT), [http://srtm.csi.cgiar.org](http://srtm.csi.cgiar.org/). Accessed 1 March 2021, (2008).
